# Exploring the unmet needs and experiences of informal caregivers of patients with end-stage kidney disease (ESKD) receiving haemodialysis – a qualitative study

**DOI:** 10.1371/journal.pone.0302525

**Published:** 2024-05-09

**Authors:** Michael Matthews, Clare McKeaveney, Helen Noble, Joanne Reid

**Affiliations:** School of Nursing and Midwifery, Queen’s University Belfast, Belfast, United Kingdom; Faculty of Medicine, Saint-Joseph University, LEBANON

## Abstract

**Background:**

Patients with end stage kidney disease (ESKD) receiving haemodialysis experience multiple symptoms, which can present physical and emotional challenges for both patients and their informal caregivers. Caregivers can experience anxiety, depression, and social isolation negatively impacting their overall wellbeing and resulting in caregiver burden. The needs of this group of caregivers have been largely neglected, with little emphasis placed on supportive interventions that might assist and support them in their caring role.

**Aim:**

The aim of this study Is to explore the unmet needs and experiences of caregivers of patients with ESKD receiving haemodialysis, and to determine the components of a supportive intervention.

**Design:**

A qualitative study using semi-structured interviews (n = 24) with informal caregivers. An interpretive qualitative framework was employed to generate a rich understanding of the unmet needs and experiences of caregivers. Data was analysed using thematic analysis. Interviews were transcribed verbatim and data management was assisted through NVIVO version 11.

**Setting/Participants:**

Twenty-four informal caregivers were purposively recruited from two haemodialysis settings within Northern Ireland.

**Results:**

Three themes were identified: (1) The negative impact of distress, anxiety, and isolation on caregivers due to their caregiving responsibilities (2) Inadequate information and knowledge about the complexities of renal care (3) The benefits of spiritual beliefs, stress management and peer support in relieving the caregiving burden.

**Conclusions:**

Caregivers of patients with ESKD receiving haemodialysis are at increased risk of physical and psychological distress and burden arising from their caregiving role. The unpredictable nature of ESKD and haemodialysis treatment negatively impacts the caregiver experience and adds to the challenges of the role. The information needs of caregivers are not always adequately met and they subsequently lack appropriate knowledge, skills, and guidance to assist them in their caregiving role. Supportive interventions are essential for caregivers to enhance their capability to deliver effective care and improve their quality of life.

## Introduction

Chronic kidney disease (CKD) has been recognised as a leading public health problem worldwide with a high economic cost to healthcare systems [1]. CKD progresses along a five-stage trajectory with Stage 5 being termed “end stage kidney disease” (ESKD) [2]. It occurs when the glomerular filtration rate of the kidney is less than 15ml/min, and renal replacement therapy is required in the form of either haemodialysis, peritoneal dialysis, or kidney transplantation [3]. Haemodialysis is the most common treatment modality [4]. While technological advances in the delivery of haemodialysis treatment have contributed to the improved survival of patients, quality of life of these patients is still much lower than that of the general population [5]. The treatment burden of patients undergoing haemodialysis is associated with adverse clinical and patient reported outcomes. These include non-adherence to medication regimens, and a higher risk of hospitalisation and mortality [6,7]. In addition, a large percentage of patients have a high co-morbidity burden and increased risk of physical, cognitive, and psychological deterioration [8,9] which negatively impacts their ability to self-care [10]. The increasing frailty and dependence on others experienced by this cohort of patients means that they are increasingly reliant on informal caregivers to help manage this debilitating condition and support them in their everyday lives [11].

Informal caregivers (hereafter referred to simply as “caregivers”) are unpaid volunteers such as family members, close friends or neighbours who provide physical, emotional, financial, and social care for an individual with a chronic illness or disability [12]. Previous research has indicated that tasks carried out by caregivers can include assistance with personal care needs, support with mobility, medication management, preparation and management of dietary and fluid requirements, monitoring for complications and provision of emotional and social support [13,14]. Caregivers of patients with ESKD undergoing haemodialysis may have their own health and social care needs and in addition may be fearful, feel vulnerable, experience conflict with their other roles and responsibilities, feel overwhelmed by their responsibilities and report unmet needs [15–17] The challenges of informal caring for patients with ESKD receiving haemodialysis often negatively impacts caregivers’ physical and psychological wellbeing, employment opportunities and social lives [18].

Despite growing recognition of the burden and adverse effects on caregivers in the provision of care for individuals receiving haemodialysis, there has been consistently little acknowledgement of their unmet needs and experiences [19]. In addition to this poor evidence-base, there is no acknowledgement of their role or specific requirements in guidance produced by the Kidney Disease Improving Global Outcomes (KDIGO) Controversies Conference [20], National Institute for Health and Care Excellence (2020) [21] or Renal Association Guidelines (2019) [22]. The negative impact of the responsibilities on this cohort of caregivers, coupled with the lack of support and dearth of evidence relating to their needs and requirements, underscores the need to understand their experiences and unmet needs, thus facilitating the development of a supportive intervention. A pilot randomised controlled trial involving family caregivers of patients with chronic kidney failure choosing not to be treated by dialysis or transplantation showed that an enhanced psychosocial program, significantly reduced caregiver burden and anxiety [23]. In the development of a supportive intervention for caregivers of patients with ESKD undergoing haemodialysis, stakeholders should take account of the expressed support needs and experiences of caregivers highlighted in this study. The aim of this study is to explore the experiences and unmet needs of caregivers of patients with ESKD receiving haemodialysis and how best they might be supported in their informal caregiving role.

## Methods

### Design

This was a qualitative study based within two renal units in Northern Ireland which in combination provides haemodialysis for approximately 140 patients. Patients undergoing home haemodialysis, which may involve a higher caregiver burden were not included in this research. Further research focusing specifically on this subgroup of caregivers is needed to determine how best to support this under-represented group. A broad interpretivist approach was adopted as the data collected from caregivers needed to be subjective and reflect their experiences in caring for patients with ESKD undergoing haemodialysis. The consolidated criteria for reporting qualitative research (COREQ) (Tong et al 2007) [24] were used to ensure best practice for reporting this study.

### Participants

Purposive sampling was employed. Senior nurses who acted as clinical gatekeepers provided patients with a caregiver a covering letter detailing the aim and objectives of the study and a copy of the participant information sheet, so that it could be given to the caregiver. A contact number was also provided allowing potential participants to contact the researcher if they had any queries regarding the study, and to inform the researcher if they were willing to participate. The inclusion criteria included participants aged 18 years or over, who were able to give informed consent, and who were nominated by the patient as their main carer and caring for a patient living at home. Caregivers were excluded if they were unable to give informed consent, were not the primary caregiver, were caring for a patient with a functioning kidney transplant and patients being cared for in an inpatient setting.

### Data collection

Semi-structured interviews were carried out by the corresponding author, MM, a PhD student, in the informal caregivers’ homes using an interview schedule designed by the research team as a prompt of key issues to explore (Table 1). To enhance validity of the interview schedule, four pilot interviews were completed before exposing it to caregivers. Written informed consent was obtained before each interview and the interviews were recorded digitally. Data was collected between 23^rd^ April 2021 and 09^th^ September 2021. Data collection was completed when data saturation was reached, and no new information or themes were being uncovered [25]. Field notes were not recorded.

**Table 1:** Participant interview schedule.

Experiences associated with informal caregiving	
**Interviewer:** Tell me about your caring role? For example, tell me about the person you care and what you do for them on a daily basis?	**Prompts:** Specific tasks for example personal care needs, medications, food and fluid requirements, monitoring loved one’s well-being, liaising with healthcare professionals, managing hospital appointments, how many hours per day you provide care, age of person caring for.
**Interviewer:** Tell me how your loved one responded to being diagnosed with ESKD and commencing haemodialysis?	**Prompts:** Does the patient talk about their condition and the need to attend hospital three times a week, does the patient expects you to take over and manage their condition or do they assume some responsibility for their own well-being.
**Interviewer:** What are your experiences caring for your relative/friend?	**Prompts:** Desire to provide good care, feelings of burden, stress, confusion, hopelessness, overwhelmed (physical and psychological considerations), relationship with partner/friend.
**Interviewer:** Can you describe the positive aspects of your caring role?	**Prompts:** Sense of achievement, personal growth and development, feeling of giving something back to someone, personal satisfaction.
**Interviewer:** What new skills do you think you have acquired in carrying out your caring role, and where did you learn these skills?	**Prompts:** Being organised, time management, flexibility, good communication skills, empathetic understanding, and adaptability to changing situations. Healthcare professionals (hospital/community based), peers, family, friends, internet, books.
**Interviewer:** How has the caring role impacted on your own life and that of other close family members?	**Prompts:** Changes in family relationships, personal/professional life balance, restrictions in social life.
**Interviewer:** What challenges or difficulties might you encounter when carrying out your caring duties?	**Prompts:** Assisting with daily care needs, care needs on return from dialysis, getting opportunity to speak with staff in dialysis unit/GP surgery, dealing with complications.
**Interviewer:** How do you cope with the challenges associated with your caregiving role?	**Prompts:** Speak with other family members, seek professional help, make time for yourself.
**Interviewer:** Looking back at the duration of you caring role, can you tell me if you have seen a change in the level of care you are required to provide?	**Prompts:** Changes in state of health of patient, changes in physical and psychological demands.
**Supportive Mechanisms**	
**Interviewer:** Can you tell me about any types of support/assistance you receive?	**Prompts:** Formal support from health and social services, GP, hospital consultants (renal or other), online support services, informal support (friends, neighbours, family support groups, other renal informal carers), support groups.
**Interviewer:** Focusing on the support from healthcare professionals, can you outline to me which members of the healthcare team provides which support?	**Prompts:** Various members of the MDT team
**Interviewer:** Tell me about any specific information or advice about caring for someone with ESKD receiving haemodialysis you have received?	**Prompts:** What was most helpful, what was least helpful? What information would you have liked, was the information/advice easily available/accessible,
**Interviewer:** Can you outline to me if you feel you have a good understanding of the needs of your loved one so you can care for them properly.	**Prompts:** Physical, psychological, and emotional care need requirements.
**Interviewer:** Can you outline to me anything you do to care for yourself or provide relaxation?	**Prompts:** Things to manage stress such as meeting friends, sitting service, help from other family members, smoking, alcohol, excessive eating.
**Interviewer:** Thinking back to when you started your caring role, what would you have found useful or would have been of benefit to you at this time?	**Prompts:** From experience what would help future carers, assist them in their caring role. Practical care needs (medication, diet and fluids), psychological/emotional support (strategies to manage stress, anxiety, depression, feelings of despair), social support (useful resources, support mechanisms), and spiritual support
**Interviewer:** If support was to be offered to informal carers maybe in terms of a structured programme, how do you think it should be delivered?	**Prompts:** face-to-face, telephone, DVD, web-based / combination of these methods, written information, individually or as a patient/carer dyad.

### Ethics

Ethical approval was granted by the West of Scotland Research Ethics Service (20/WS/0056) on 3^rd^ April 2020, who granted permission for the study to be completed in Northern Ireland. Sponsorship for the study was obtained from Queen’s University, Belfast. Governance approval was gained from the Research and Governance Offices, Northern Health and Social Care Trust (NT20-2740230-04) on 1^st^ July 2020, and Belfast Health and Social Care Trust (20003HN-SS) on 16^th^ April 2021. The study was conducted in compliance with Good Clinical Practice Guidelines [26].

### Analysis

Data from the recorded semi-structured interviews was transcribed verbatim, checked for accuracy against the original audio files and read several times [27]. A thematic content analysis was carried out as outlined by King and Horrocks (2010) [28]. This method of analysis defines themes as recurrent and distinctive features of participants’ accounts, characterising perceptions and/or experiences which the researcher sees as relevant to the research question. This approach was chosen as it aligns with the interpretivist approach of the study, whereby the researcher aimed to interpret and understand caregivers’ experiences of caring for patients with ESKD receiving haemodialysis and gain the views of healthcare staff regarding the development of a supportive intervention. During the process of thematic analysis, data was analysed to identify common themes and group recurring themes occurring in the data. Data coding was completed by MM and checked by the co-authors. Data management was assisted by using NVivo version 11 (QSR 2015) [29]. As data analysis could have reflected personal bias, reflexivity increased the credibility and rigor of the research findings. Rigor was maintained ensuring that all conclusions made in the research were dependable and transparent. An audit trail was kept detailing problems encountered during the study and measures put in place to resolve these issues [30].

## Results

**Fig 1:**
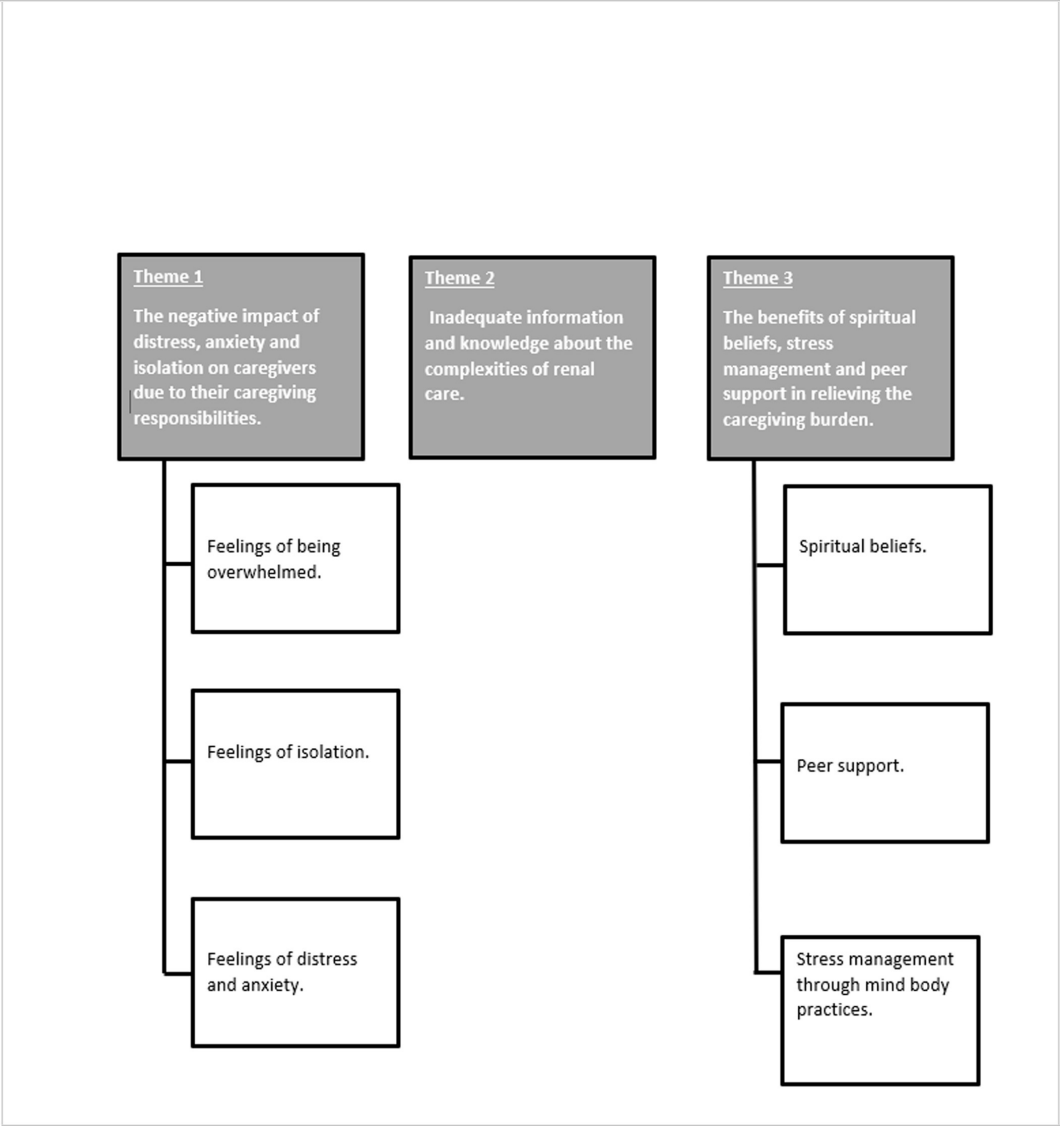
Thematic schema showing connection and relationship between themes and sub-themes.

Twenty-four semi-structured interviews were conducted with caregivers between April 2021 and September 2021. Participants age ranged from 35–86 years (mean age = 66). Fourteen female and ten male caregivers, all of white ethnicity participated in the interviews which lasted from 55 to 95 minutes in duration (mean = 70 minutes). In-person face-to-face interviews (n = 15) and virtual interviews (n = 9) were completed using videoconferencing software. These latter were offered to allow social distancing in response to the COVID-19 pandemic. All participants approached participated in the research. No repeat interviews were carried out, and no transcripts were returned to participants for comment or correction. Additional details about the participants are presented in Table 2. Three overarching themes were generated from the interview data and are presented below. A thematic schema has been included to visually represent the connection and relationship between themes and sub-themes Fig 1. Thematic schema showing connection between themes and sub-themes.

**Table 2:** Profile of informal caregivers who participated in the semi-structured interviews.

Participant identification	Gender of informal caregiver	Ethnicity	Age of informal caregiver	Informal caregiver relationship to patient	Informal caregiver and patient living together	Occupation of informal caregiver	Interview type
IC-001	Female	White	69	Friend	No	Retired	Face to face
IC-002	Female	White	57	Daughter	Yes	Domestic Assistant	Face to face
IC-003	Female	White	55	Mother	Yes	Housewife	Face to face
IC-004	Female	White	51	Daughter	Yes	Unemployed	Video conferencing
IC-005	Female	White	75	Wife	Yes	Retired	Video conferencing
IC-006	Female	White	35	Friend	No	Carer	Video conferencing
IC-007	Male	White	61	Husband	Yes	Civil servant	Face to face
IC-008	Female	White	74	Wife	Yes	Retired	Video conferencing
IC-009	Female	White	77	Wife	Yes	Retired	Video conferencing
IC-010	Male	White	61	Husband	Yes	Unemployed	Face to face
IC-011	Male	White	78	Partner	Yes	Retired	Video conferencing
IC-012	Female	White	80	Wife	Yes	Retired	Face to face
IC-013	Male	White	49	Brother	No	Business Owner	Face to face
IC-014	Female	White	41	Granddaughter	Yes	Nurse	Video conferencing
IC-015	Male	White	34	Son	Yes	Unemployed	Face to face
IC-016	Female	White	56	Wife	Yes	Classroom assistant	Face to face
IC-017	Male	White	32	Son	Yes	Taxi driver	Face to face
IC-018	Female	White	59	Daughter	Yes	Bookkeeper	Face to face
IC-019	Male	White	67	Husband	Yes	Retired	Video conferencing
IC-020	Female	White	46	Daughter	No	Teacher	Video conferencing
IC-021	Male	White	64	Daughter	No	Retired	Face to face
IC-022	Male	White	74	Husband	Yes	Retired	Face to face
IC-023	Male	White	54	Son	Yes	Unemployed	Face to face
IC-024	Female	White	86	Wife	Yes	Retired	Face to face

## Theme 1. The negative impact of distress, anxiety, and isolation on caregivers due to their caregiving responsibilities

Caregiving had a negative impact on the emotional and psychological well-being of the caregivers’ participating in the study. They reported that they felt overwhelmed by the responsibilities of their care giving role, that they had lack of time for themselves, and that they experienced feelings of inadequacy and isolation. This first theme was hence comprised of three sub-themes which are explored below: feelings of being overwhelmed, feelings of isolation and feelings of distress and anxiety.

### Sub-theme 1. Feelings of being overwhelmed

Firstly, caregiving was shown to have a negative impact on the participants’ emotional and psychological well-being as they tried to juggle personal, household, and professional roles, which led to them feeling overwhelmed. These competing demands for their time and energy resulted in caregivers being unable to fulfil their many roles to the best of their ability and it was difficult if not impossible for caregivers to make time for themselves.

“You don’t get a chance to sit down all evening… prepare dinner, get pills, get her ready and assist her to bed, and then you have to prepare for the next day, while trying to make time for your husband and children. My husband is very understanding …. and because you are so exhausted, I feel I’m not giving things my 100%, but I am giving my best to everything . . . my husband, my mother my children, my job and of course I’m last, I’ve no time to call my own….” (IC-020)

### Sub-theme 2. Feelings of isolation

Secondly the issues of isolation that arose in terms of the significant time commitment required by caring frequently led to participants describing the need to be constantly attentive to the person being cared for. The participants reported feelings of isolation and loneliness connected to the disconnection from family members and friends which further contributed to their emotional and psychological distress.

*“It’s impossible to organise anything, the caring role just consumes your whole life…, he is constantly on my mind, first thing in the morning, last thing at night*. *We might plan to do something in the short window we have outside of dialysis days and then on the days he is not for dialysis he is so weak and tired, that plans have to be cancelled*. *It could all get you down very easily”. (IC-08)*.

### Sub-theme 3. Feelings of distress and anxiety

Participants expressed psychological distress and anxiety due to fear and uncertainty about the future care of their loved one. Caregivers recognised their loved one had no clear illness trajectory and little guidance from healthcare professionals which they found frightening. Caregivers were under no illusion, as time goes on, they too will experience decline in health, given the unrelenting nature of their caregiving role resulting in a lot of unanswered questions, adding to their anxiety.

“*I worry about if the time comes that I am no longer able to care for him…*.*and with me getting older and having my own health problems…*. *that worries me very much*, *who will look after him and who will care for him*? *he is so very dependent on me*” (IC-003).

Caregivers were particularly distressed when they witnessed their loved one experiencing the adverse effects of dialysis such as extreme lethargy, nausea, and muscle cramps. In the meantime, they could do little to alleviate the suffering.

*“I feel so sorry for [name] my wife when she returns home from dialysis*, *she is just completely washed out and fit for nothing*, *and this all causes extra stress*, *deciding if you need to contact the doctor or even the ambulance*. *I have no background in anything medical*, *I don’t understand why there is no-one could speak to you even to say yes or no …*. *give you advice on this”* (IC-013).

A further pressing concern that caused distress and anxiety for many caregivers was the financial implications of their caring role. Caregivers found financial support difficult to access, dependent on complex eligibility criteria and associated with restrictions on employment which further limited their ability to manage the financial burden.

*“I really feel carers should be financially rewarded*, *I had to give up a well-paid job to meet the care needs of my father*, *after the completion of laborious forms I am now in receipt of some benefits*, *but it in no way compensates my previous wage*. *I am continually dipping into my savings”*. (IC-023).

Poor lifestyle choices such as comfort eating and not taking regular exercise were symptomatic of the participants’ response to stressful caregiving situations. In the longer-term caregivers were aware that these behaviours may have a detrimental effect on their well-being and further impact their ability to provide care. In response to feeling overwhelmed by these feelings, participants expressed an interest in being able to access a web-based health promotion link and/or being signposted to a facility where they could engage in activities to promote physical activity and healthier eating choices.

*“I know I’m not doing myself any favours and am aware of the problems that could present resulting from a sedentary lifestyle and unhealthy eating*. *It’s difficult to leave here to take part in a fitness class*. *What would be good would be a link where you could log on to get information on healthier food choices and even an exercise or fitness session which you could take part in at home”*.

## Theme 2. Inadequate information and knowledge about the complexities of renal care

As the participants attested, taking on new practical roles and responsibilities proved challenging due to a lack of information and knowledge. Such responsibilities came from assisting with personal care needs and managing food and fluid requirements, to the administration of medications and additional household tasks which caused caregivers to feel overwhelmed. These issues are explored across this theme through the following four core elements: inadequate medication management knowledge, difficulties in providing personal care needs, inadequate knowledge of the renal diet and poor knowledge of the complexities of renal care.

Firstly, participants reported that medication management was a complex task requiring physical and cognitive effort to order, collect, organise store and administer medications correctly. These tasks imposed substantial responsibility and practical difficulties on caregivers, adding significantly to their level of burden such as concern that they could forget to administer medication which might negatively impact on the health of their loved one.

*“Trying to manage and administer tablets is so challenging*, *he is on so many tablets for renal*, *diabetes*, *heart failure*. *My daughter suggested a blister pack*, *but that wouldn’t work with the phosphate binders to be taken at specific times and the changing dose of warfarin”*. *I have to write myself notes to make sure I don’t forget to give tablets at the prescribed time*. *{Name} hates taking tablets and would never ask for them but then his memory isn’t always the best anyway”*. (IC-006).

Secondly caregivers explained how personal care and toileting were particularly stressful caregiving tasks. Participants wanted reassurance sufficient to know that what they were doing was correct, particularly in terms of protecting skin integrity. The mobility of patients was often reduced, and many had to spend long periods resting in bed. One participant (IC-011) repeatedly emphasised having received no guidance on skincare and described how:

“*You hear so much about people developing pressure sores*, *especially on their bottom*, *I’m not even sure what to look for*, *or what I could do to help prevent this happening*, *some information about this would have been useful*, *but it was never forthcoming*, *I just do what I think is best”*. (IC-011).

Physical changes to the home environment such as the installation of a stair lift, a wet room or the provision of additional toileting equipment were felt by participants to be contributions that would assist them in their caregiving role. While some caregivers were awaiting an assessment by an occupational therapist, most caregivers had received no input from occupational therapy or were unaware of the support that these healthcare professionals could offer them. The participants also reflected a need for awareness and knowledge of what support voluntary organisations could provide and how contact could be made with these organisations.

“*We are continually struggling at home*, *neither of us are getting any younger and (name) is just losing the power in his legs*. *My GP eventually made a referral to the occupational therapist*, *but God knows how long the waiting list is*, *so in the meantime you just have to struggle on*. *The GP surgery or renal unit told me they couldn’t provide a wheelchair but didn’t say where I could get one*. *Then just by chance a friend of a friend said you get them from the British Red Cross*, *you just have to make a donation*, *why could nobody have told me that*, *I was distracted didn’t know what I was going to do*, *but thank God*, *they sorted me out”*.

Thirdly caregivers reported that the provision of personal care and medication management was further compounded by frustration with the different components of the renal diet. Participants had little knowledge of the purpose of the diet and the overall significance of the diet in managing the patient’s condition. Many caregivers were also supporting patients with other co-morbid conditions such as diabetes and heart failure, conditions which also necessitated dietary and fluid restrictions. The participants reported that their loved ones sometimes brought home information sheets on how to manage dietary and fluid requirements, but others had never received such literature. Moreover, the caregivers were unable to confirm if this meant their loved ones were not reviewed by the dietician or if they discarded or left the information sheets behind them.

*“I can understand dietary and fluid restrictions for the diabetes and heart failure*, *but for kidney disease I have no idea*, *how can you deal with all these special requirements when preparing one single meal*. *I’m at a loss as to what the information sheet means*, *you end up bombarded with sheets on diet and fluids and just end up so confused*, *the dietician really needs to make contact with me*, *so I have it clear in my mind what I’m supposed to be doing*, *I have a lot of questions”*. (IC-011).

In terms of the fourth and last element of this theme that addresses caregivers’ lack of knowledge, participants demonstrated limited understanding of some of the complexities of renal care such as how to care for a fistula or haemodialysis catheter. Fistulas and haemodialysis catheters are essential lifelines for patients and the means by which blood is carried to a haemodialysis machine. Caregivers reported that they realised the importance of keeping the site of the fistula or haemodialysis catheter clean. However, they were unaware of the potential complications that might present, how to recognise these complications and what to do in an emergency if the fistula or haemodialysis catheter started to bleed. This led to additional stress.

“*I regularly check his fistula to see if it bleeds*, *but don’t know what else I should be looking for*, *I take the plasters off but am always terrified of the thing bleeding*, *what would I do then*, *I would have to contact an ambulance”*. (IC-023).

These limitations in the face of the complexity of renal care were further exacerbated by the limited opportunities to speak to medical or nursing staff about fluctuating patient symptoms. The caregivers were often left worrying about the seriousness of symptoms and felt that they had to learn to identify when they could deal with these problems themselves or whether they had to seek professional assistance.

*“This all causes extra stress*, *it’s just a stress*, *deciding if you need to contact the doctor or even the ambulance*. *I have no background in anything medical*, *I don’t understand why there is no-one could speak to you even to say yes or no …*. *give you advice on this”* (IC-013).

Furthermore, caregivers reported the need for tailored educational programs involving input from healthcare professionals using language which was easy to comprehend. When a loved one starts haemodialysis caregivers may require information related to the haemodialysis treatment, whereas over-time the information needs may change to manage the complications arising from the haemodialysis treatment. Participants concurred that there was a need for specific educational programs which would meet their ever-changing knowledge and information needs in caring for a patient with ESKD on haemodialysis.

*“The information you need to provide proper care changes all the time*, *what information was useful to me when {name} started dialysis is no longer beneficial now two years later*. *Whatever the technicalities are*, *healthcare workers need to bear in mind they are talking to the layman*, *but look*, *this is where it was*. *We stepped in*. *This is where it is*. *And now it’s doing that*. *We’re good*. *Simple language is the only way”* (1C-007).

## Theme 3. The benefits of spiritual beliefs, stress management and peer support in relieving the caregiving burden

Participants acknowledged the positive elements associated with their caring role. They highlighted the importance of adopting a positive attitude and focusing more on these positive aspects which helped them deal with challenges and stressful situations to provide ongoing motivations to sustain them in their role. The factors associated with influencing a more positive caregiving experience are discussed through consideration of three sub-themes: spiritual beliefs, peer support and stress management through mind body practices,

### Sub-theme 1. Spiritual beliefs

Participants highlighted the importance of adopting a positive attitude and focusing on the encouraging aspects of their caring role. Caregivers acknowledged the positive elements associated with informal care provision which included the development of a closer relationship with their loved one. Caregivers developed a stronger affiliation with their spiritual beliefs through faith and prayer, both of which provided ongoing motivation to sustain their caregiving role. There is no cure for ESKD, and many patients are too unwell to be considered for a kidney transplant. In this context spiritual beliefs became significant, and faith and trust provided some optimism and hope for the future. For some the sense of being part of a religious community offered caregivers a sense of belonging with several finding great comfort when people said to them “I’ll remember you in my prayers”.

*“I have a very close church family*, *there is genuine concern for each other……*, *this means a lot*. *I make the biggest effort to go to church each Sunday*, *this sustains me for the remainder of the week*. *On the odd occasion I cannot get to church there are numerous phone calls to see if everything is ok*, *that just shows how close the church family is”*. (IC-005).

### Sub-theme 2. Peer support

Participants acknowledged the valuable role of peer support providing a mechanism whereby they could receive guidance, empathetic understanding, and helpful tips from informal carers who had been/or were in a similar caregiving situation as themselves, thus helping them adjust and adapt to their informal caregiving situation.

*“In addition to the support of healthcare professionals*, *having the opportunity at the same time having the chance to speak to someone who has been through this is just as important*, *so they can share their experiences …*. *what works and what doesn’t*. (IC-019).

### Sub-theme 3. Stress management through mind body practices

Caregivers often admitted that they struggled to make time for themselves which resulted in increased stress and anxiety. They deal with this in a variety of ways including mind body practices such as mindfulness, meditation and counselling. Participants reported that such practices could help reduce some of the stress associated with informal care provision.

*“I am a long-term practitioner of meditation and mindfulness*, *which helps me deal with the stress of my caring role*, *I feel this would benefit others because without this I do not know what state I would be in*” (IC-011).

## Discussion

While the impact of informal caregiving can take its toll on informal caregivers, there are also positive aspects associated with the informal caregiving role. Firstly, participants conveyed the feeling of how being a caregiver had a negative impact on their physical, psychological, and social lives, contributing to a high level of caregiver burden. The concept of caregiver burden is multidimensional and is the result of multiple responsibilities including managing dietary and fluid requirements, medication management, dealing with haemodialysis related complications and attending to personal care requirements and household duties. But at the same time, caregivers identified positive aspects in how caregiving provided them an opportunity to strengthen connections with their loved one, and a correlating sense of fulfilment from committing to care.

The findings from the current study concur with recent systematic reviews into caregiving in a range of settings, not just the haemodialysis setting. They have identified that caregivers of patients with multimorbidity and those of patients with cancer experienced significant caregiver burden which compromised their overall health and wellbeing [31,32]. They likewise concur with those systematic reviews which have considered caregiving in a range of different renal settings including haemodialysis, peritoneal dialysis, renal transplantation and those patients who are managed conservatively [33–35]. The current study further contributes to the existing knowledge base by eliciting novel findings relating to what caregivers feel would improve their caregiving experience, whilst considering the views of healthcare professionals on the components of a supportive intervention to educate and support caregivers in their caring role.

The perceived lack of input in terms of practical, psychological, social, and emotional support from healthcare professionals and family members or friends further exacerbated the difficulties caregivers experienced. Like this study, family caregivers caring for patients with heart failure have also identified poor support from healthcare professionals as a major challenge. This had a negative impact on the ability of family members and friends to provide safe care while coping with the varied demands of the caregiving role [36]. Difficulty in obtaining a broad range of information needs from healthcare professionals was identified in our study which contributed to the uncertainty and stress experienced by caregivers. There was a need for care related information as well as information about services and available support i.e., in terms of finances. This phenomenon was akin to the experience of caregivers in other countries where dissatisfaction with healthcare providers in terms of information provision resulted in greater caregiver burden and a subsequent reduction in caregivers’ psychological wellbeing [37,38].

Despite the challenges and burden experienced by caregivers in this study, findings also show dimensions to facilitate positivity and motivation to continue informal care provision. These include the development of closer caring relationships, commitment to provide care, and the opportunity to reciprocate care. Spiritual beliefs also constituted an important motivating factor for informal caregiving. Previous research in the field of dementia and cancer concurs that reciprocity, family duty and filial obligation are frequently cited motives for informal care provision [39–41]. Across different cultures such as South Africa, China and Brazil the reasons for providing informal care are similar to filial obligation and reciprocity emphasising commonalities across different cultures [42–44]. The increasing evidence to indicate the benefits of positive aspects of informal caregiving further highlights the need for further research into developing a supportive intervention which includes strategies to foster a more positive caregiving experience. The findings from this research will be used to inform future adaptation of practice and supportive intervention development targeting informal caregiver’s specific needs.

## Strengths and limitations

The strength of this study is its account of the phenomenon of caregivers caring for a person with ESKD receiving haemodialysis, through the views and experiences of carers using semi-structured interviews. The research has provided a rich description of their daily experiences and unmet needs, highlighting the challenges and difficulties as well as the more positive aspects of informal care provision in this cohort of patients. This study provides a foundation upon which to develop the components of a supportive intervention. However, despite the rigorous research design, all participants were from a white ethnic origin. It was not possible, therefore, to compare different ethnic groups which limit the generalisability of the findings. The participants came from two haemodialysis settings within Northern Ireland. This constrained the transferability of findings to populations outside Northern Ireland given the diversity of the delivery of healthcare in other settings and cultures.

## Conclusion

This qualitative research study highlights the high level of burden, unmet practical needs and lack in psychological support given the complexity of experiences for caregivers of patients with ESKD undergoing haemodialysis. The expectations and demands of caregivers of this patient group continue to grow as the numbers of people with multiple co-morbidities accepted unto haemodialysis increases year on year. The findings from this study identifies opportunities in terms of practical, psychological, and social support. In doing so healthcare providers might now have the opportunity to develop and implement a suitable intervention to facilitate a more positive caregiving experience through the provision of knowledge, skills and assistance to facilitate the demands of their caregiving responsibilities, enabling them to perform their caring role more effectively.
